# Supplementary material for: Determining the timing of pubertal onset via a multicohort analysis of growth
Source: PLoS One. 2021 Nov 18;16(11):e0260137. doi: 10.1371/journal.pone.0260137 (PMC8601458; doi:10.1371/journal.pone.0260137)
Supplement: S1 Fig — (DOCX) [file pone.0260137.s001.docx]

**
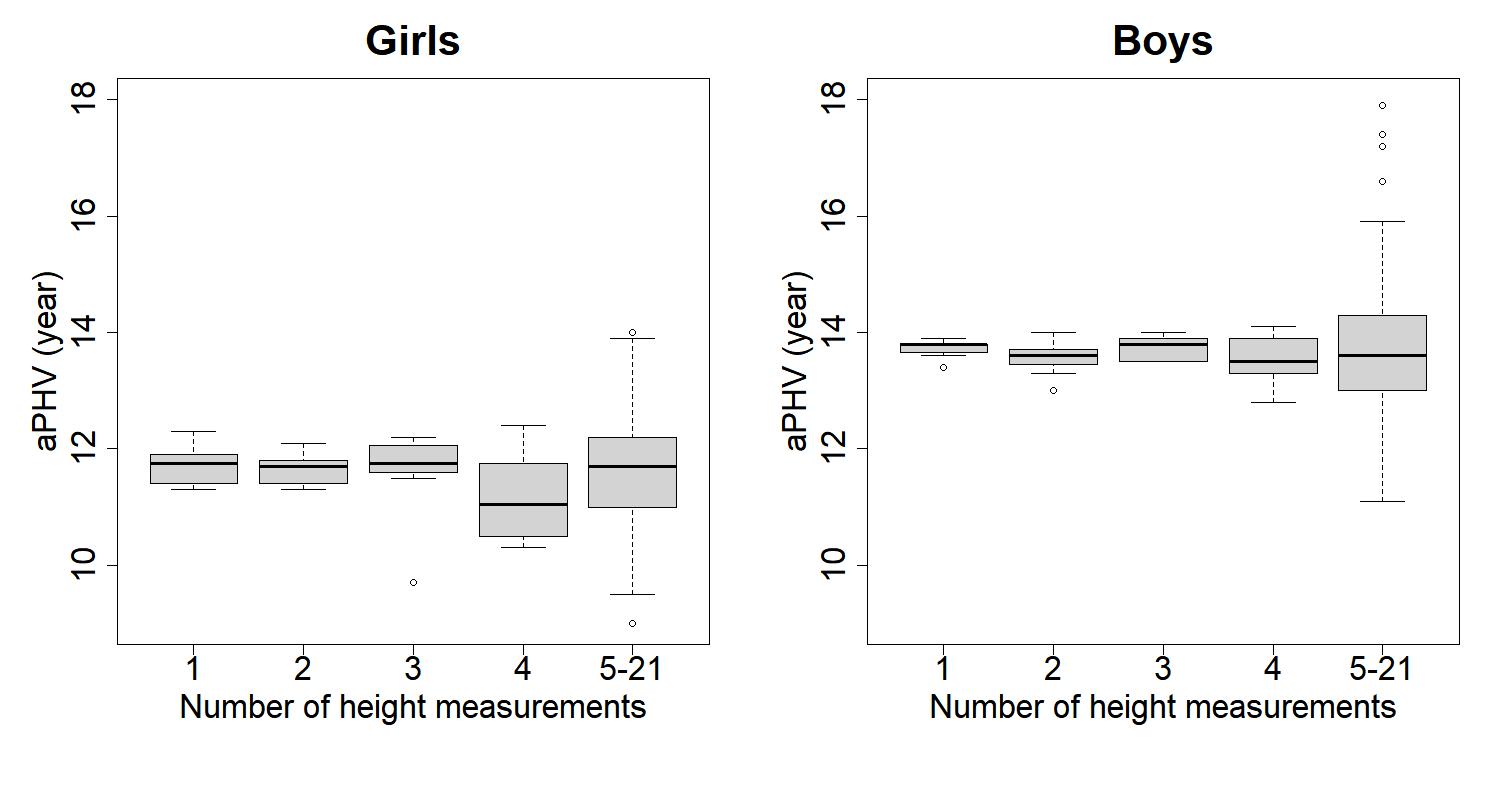
S1 Fig**. **Distributions of the aPHV values by the number of the height measurements for girls and boys.**
