# Supplementary material for: Determining the timing of pubertal onset via a multicohort analysis of growth
Source: PLoS One. 2021 Nov 18;16(11):e0260137. doi: 10.1371/journal.pone.0260137 (PMC8601458; doi:10.1371/journal.pone.0260137)
Supplement: S2 Fig — Subject-specific ages at peak height velocity (aPHV), peak height velocities (PHV) and size parameter values plotted against the observed pubertal onset intervals for girls (above) and for boys (below). Passing line is the regression line between the growth marker and the midpoint of the observed pubertal onset interval. (DOCX) [file pone.0260137.s002.docx]

**
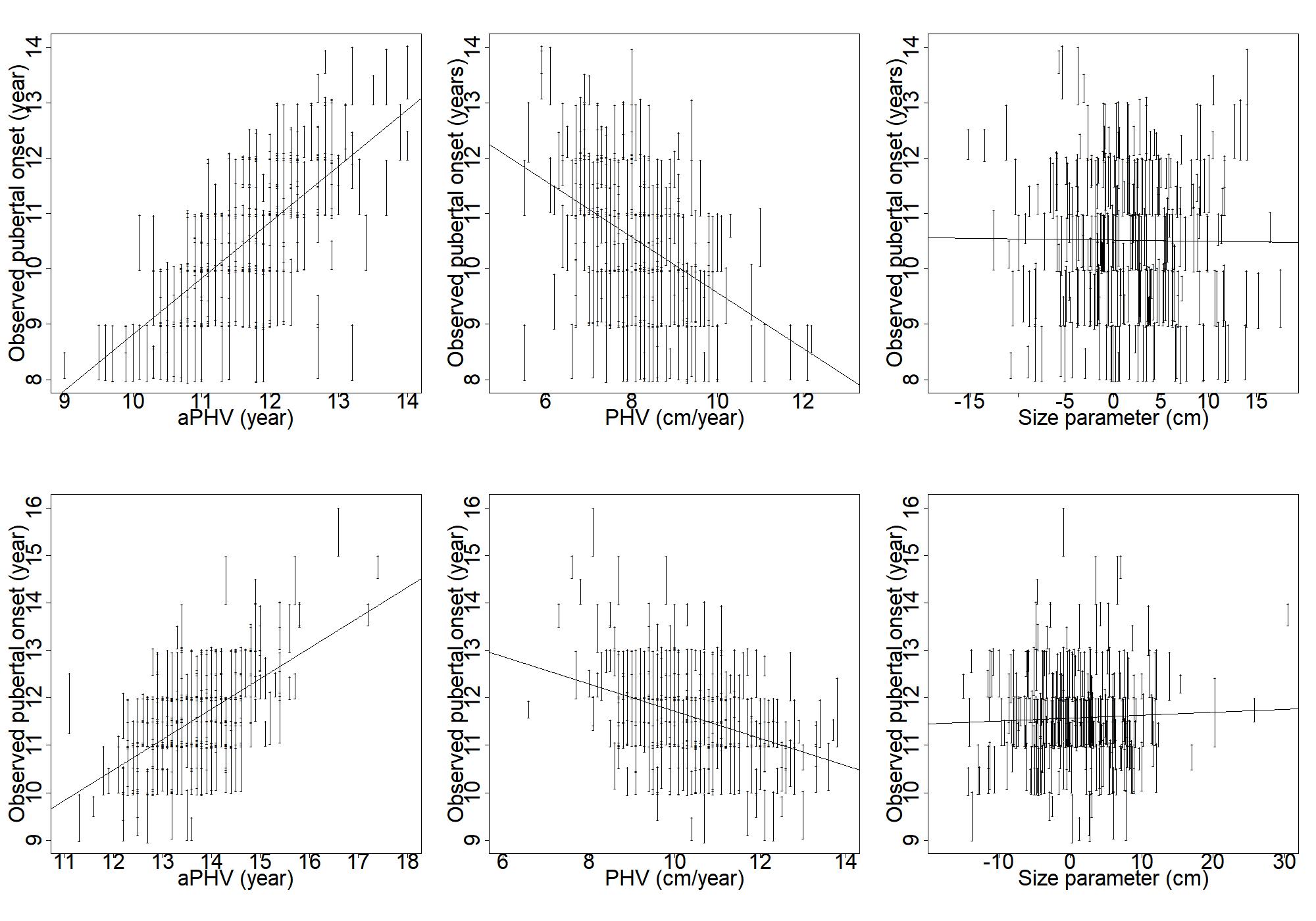
**

**S2 Fig**. **Associations between growth markers and the age at pubertal onset.** Subject-specific ages at peak height velocity (aPHV), peak height velocities (PHV) and size parameter values plotted against the observed pubertal onset intervals for girls (above) and for boys (below). Passing line is the regression line between the growth marker and the midpoint of the observed pubertal onset interval.
