# Supplementary material for: Determining the timing of pubertal onset via a multicohort analysis of growth
Source: PLoS One. 2021 Nov 18;16(11):e0260137. doi: 10.1371/journal.pone.0260137 (PMC8601458; doi:10.1371/journal.pone.0260137)
Supplement: S3 Fig — Individual aPHVs plotted against the observed pubertal onset intervals separately for normal weight and overweight girls. Passing line is the regression line between aPHV value and the midpoint of the observed pubertal onset interval. (DOCX) [file pone.0260137.s003.docx]

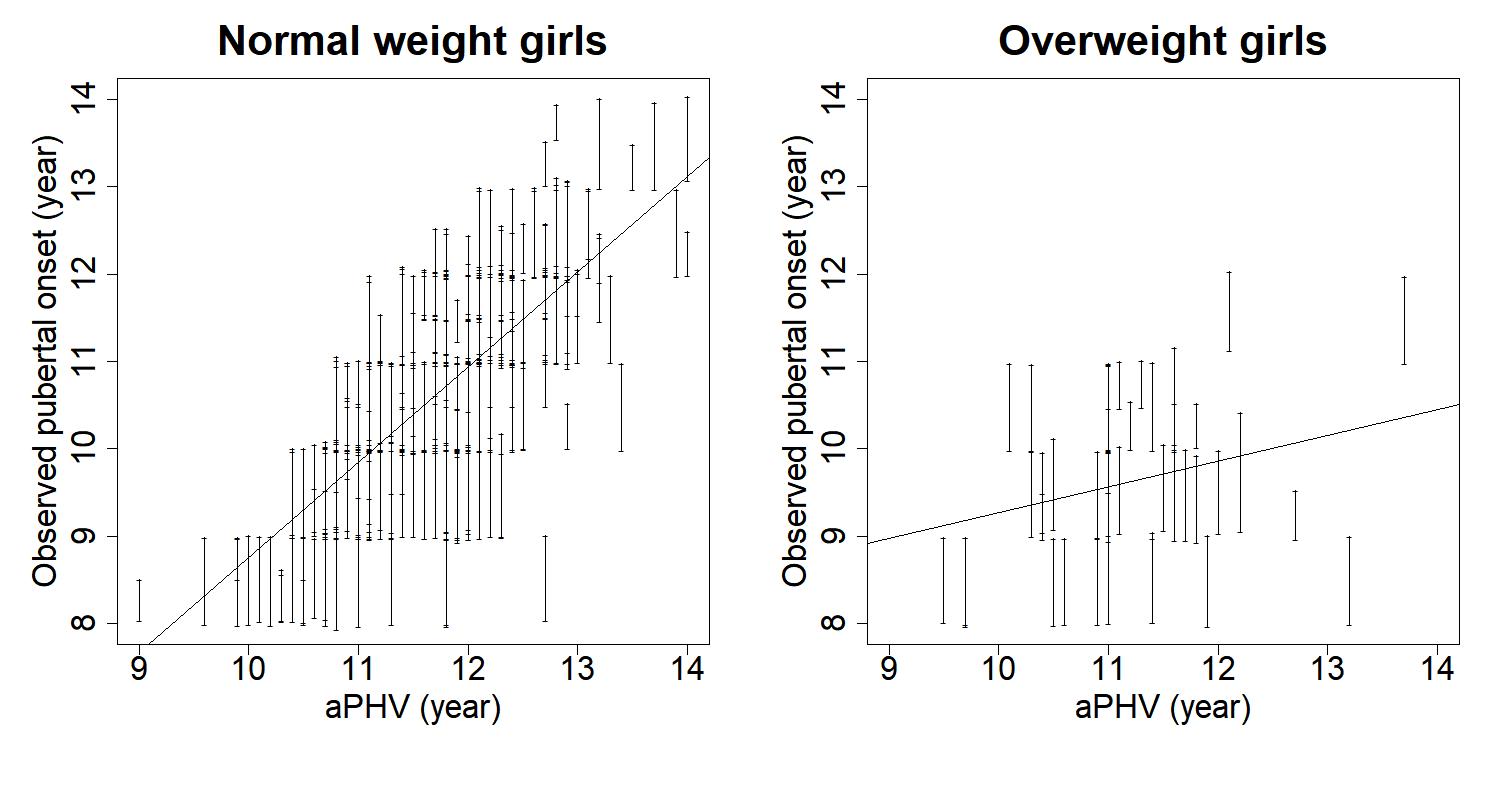
**S3 Fig**. **Association between aPHV and the age at pubertal onset for normal weight and overweight girls**. Individual aPHVs plotted against the observed pubertal onset intervals separately for normal weight and overweight girls. Passing line is the regression line between aPHV value and the midpoint of the observed pubertal onset interval.
