# Supplementary material for: Determining the timing of pubertal onset via a multicohort analysis of growth
Source: PLoS One. 2021 Nov 18;16(11):e0260137. doi: 10.1371/journal.pone.0260137 (PMC8601458; doi:10.1371/journal.pone.0260137)
Supplement: S1 Table — Parameter estimates of the time-to-pubertal onset models with standard errors in brackets, and agreement between the observed and predicted pubertal onset for simple and extended model. (DOCX) [file pone.0260137.s004.docx]

**S1 Table.** **Parameters of the time-to-pubertal onset models, with prediction results.** Parameter estimates of the time-to-pubertal onset models with standard errors in brackets, and agreement between the observed and predicted pubertal onset for simple and extended model.

|  | **Variable** | **Simple model** | **Extended model** |
| --- | --- | --- | --- |
| **Girls** | Intercept | 0.15 (1.30) | 0.30 (1.32) |
|  | aPHV | 0.94 (0.08) | 0.97 (0.08) |
|  | PHV | -0.07 (0.06) | -0.12 (0.06) |
|  | Overweight |  | 8.24 (1.94) |
|  | aPHV x Overweight |  | -0.78 (0.17) |
|  | Agreement^a^ | 68.1% | 71.0% |
| **Boys** | Intercept | 5.16 (0.92) | 4.51 (0.95) |
|  | aPHV | 0.54 (0.05) | 0.58 (0.05) |
|  | PHV | -0.10 (0.03) | -0.08 (0.03) |
|  | Overweight |  | 0.23 (0.11) |
|  | Agreement^a^ | 76.0% | 77.0% |

^a^After the 10-fold cross-validation
