# Supplementary material for: Determining the timing of pubertal onset via a multicohort analysis of growth
Source: PLoS One. 2021 Nov 18;16(11):e0260137. doi: 10.1371/journal.pone.0260137 (PMC8601458; doi:10.1371/journal.pone.0260137)
Supplement: S1 File — (DOCX) [file pone.0260137.s005.docx]

S1 File. Methodological supplementary.

**First stage: Super-Imposition by Translation And Rotation (SITAR) growth curve model**

The basic form of the SITAR model is

$$y_{i}(t)=\alpha_{0}+a_{i}+\sum_{k=1}^{K} \delta_{k}B_{k}\left( \frac{t-\beta_{0}-b_{i}}{e^{-\left( \gamma_{0}+c_{i} \right)}} \right)+\varepsilon_{i}(t),$$

where $y_{i}(t)$ is the observed height of the *i*th child at mean-centered age *t*. Here $\alpha_{0}$, $\beta_{0}$, and $\gamma_{0}$ are fixed-effect parameters of size, timing and intensity, respectively; the $a_{i}$, $b_{i}$ and $c_{i}$ are corresponding random effect parameters for child *i*; $B_{k}(.)$ represents the value of the *k*th spline basis function for a piecewise cubic spline at time *t* with corresponding fixed regression coefficient $\delta_{k}$; and $\varepsilon_{i}(t)$ are independent normally distributed errors. The random effects $a_{i}$, $b_{i}$ and $c_{i}$ were assumed to be independent between individuals and have normal distribution with a mean of zero.

The piecewise cubic spline curve of the model was fitted with a natural cubic regression spline. Knots were placed at equally long intervals. Age was log-transformed as recommended by Cole et al. [1] and this was also verified in our data by an investigation of BIC and deviance. The number of knots (3–8) was chosen by the Bayesian Information Criterion (BIC) [2] after fitting several models with different starting values. Models with different starting values were fitted to solve convergence problems, which are common with nonlinear models, and to reach a stable solution. [3] The BIC and deviance yielded model with logarithmic age-transformation and five knots for girls and six for boys.

To investigate the cohort differences in growth, cohort-specific fixed-effect parameters were added into the model. For girls we set

$$y_{i}\left( t \right)=\alpha_{0}+\alpha_{S}+a_{i}+\sum_{k=1}^{5} \delta_{k}B_{k}\left( \frac{t-\beta_{0}-\beta_{S}-b_{i}}{e^{-\left( \gamma_{0}+\gamma_{S}+c_{i} \right)}} \right)+\varepsilon_{i}\left( t \right),$$

and for boys we set

$$y_{i}\left( t \right)=\alpha_{0}+\alpha_{S}+\alpha_{B}+a_{i}+\sum_{k=1}^{6} \delta_{k}B_{k}\left( \frac{t-\beta_{0}-\beta_{S}-\beta_{B}-b_{i}}{e^{-\left( \gamma_{0}+\gamma_{S}+\gamma_{B}+c_{i} \right)}} \right)+\varepsilon_{i}\left( t \right),$$

where *t* stands for the mean-centered logarithm of age, subscript *S* stands for the STRIP study and subscript *B* stands for the Boy cohort study. The DIPP study was used as the reference cohort.

**Second stage: Time-to-pubertal onset model**

For girls, the extended model identified was:

$$T_{i}=\mu+\delta_{1}\mathrm{aPHV}_{i}+\delta_{2}{PHV}_{i}+\delta_{3}{BMI\_group}_{i}+\delta_{4}{aPHV_{i}\times BMI\_class}_{i}+\sigma\varepsilon_{i},$$

and for boys:

$$T_{i}=\mu+\delta_{1}\mathrm{aPHV}_{i}+\delta_{2}{PHV}_{i}+\delta_{3}{BMI\_class}_{i}+\sigma\varepsilon_{i}.$$

$T_{i}$ is treated as survival time and includes censoring. The random errors $\varepsilon_{i}$ were assumed to be independent and to follow a standard normal distribution with mean zero and unit variance so that $\sigma^{2}$ represents the residual variance.

References

1. Cole TJ, Donaldson MD, Ben-Shlomo Y. SITAR—a useful instrument for growth curve analysis. Int J Epidemiol. 2010;39: 1558-1566. doi: 10.1093/ije/dyq115.

2. Schwarz G. Estimating the dimension of a model. Ann Stat. 1978;6: 461-464. doi: 10.1214/aos/1176344136.

3. Lindstrom MJ, Bates DM. Nonlinear mixed effects models for repeated measures data. Biometrics. 1990;46: 673-687.

stylefix
