# Supplementary material for: Determining the timing of pubertal onset via a multicohort analysis of growth
Source: PLoS One. 2021 Nov 18;16(11):e0260137. doi: 10.1371/journal.pone.0260137 (PMC8601458; doi:10.1371/journal.pone.0260137)
Supplement: Attachment — Submitted filename: Response to Reviewers.docx [file pone.0260137.s006.docx]

**Response to Reviewers**

**Reviewer #1:** The authors present SITAR analysis of growth relative to pubertal analysis using three Finnish cohort studies—the Type 1 Diabetes Prediction and Prevention (DIPP) Study, the Special Turku Coronary Risk Factor Intervention Project (STRIP), and the Boy cohort. The authors conclude that their novel modeling approach provides an opportunity to evaluate Tanner breast/genital stage–based pubertal onset timing in cohort studies including longitudinal data on growth but lacking pubertal follow-up.

The study represents an important contribution to puberty research but the authors may have overstated the importance of their findings on line 458 and 459 as 'immediate clinical relevance and applicability'. Replication of these results is other dataset is warranted before such a generalization can be made. Perhaps they could suggest that this method is promising and with replication in other cohorts ...

*Thank you for the essential remark. We changed the sentence on lines 477-479 (in a manuscript with tracked changes) as “The determination of pubertal onset timing based on pubertal growth markers is promising method to facilitate the determination of the timing in adolescent cohort studies, and with replication in other cohorts this may have immediate clinical relevance and applicability.”*

**Reviewer #2:** Good manuscript takes up available data and utilizes models to draw conclusions well written but if the models used like SITAR can be explained a bit it would be good for the average reader. Also the data has not been released due to privacy restrictions can that be explained more.

*Thank you for the relevant comment. We have now clarified the SITAR modeling in the “First stage: Super-Imposition by Translation And Rotation (SITAR) growth curve model” section. We also added a reference to the Supporting S1 File in the end of the SITAR model section and in the end of the “Second stage: Time-to-pubertal onset model” section. S1 File includes the full technical details of the modeling.*

*We also revised our Data Availability statement as “The dataset supporting the conclusions of this article were obtained from the DIPP, STRIP and Boy cohort studies. The dataset comprises health related participant data and their use is therefore restricted under the regulations on professional secrecy (Act on the Openness of Government Activities, 612/1999) and on sensitive personal data (Personal Data Act, 523/1999, implementing the EU data protection directive 95/46/EC). Due to these legal restrictions, the data from this study cannot be stored in public repositories or otherwise made publicly available. However, data access may be permitted on a case by case basis upon request only. Data sharing outside the research groups is done in collaboration with DIPP, STRIP and Boy cohort groups and requires a group-specific data transfer agreements (DTA). Investigators can submit an expression of interest to the chairman of the DIPP steering committee (Prof Riitta Veijola, University of Oulu, Oulu, Finland. Email: riitta.veijola@oulu.fi), to the chairman of the STRIP steering group (Prof Olli Raitakari, University of Turku, Turku, Finland. Email: olli.raitakari@utu.fi) and to the chairman of the Boy cohort steering group (Prof Jorma Toppari, University of Turku, Turku, Finland. Email: jorma.toppari@utu.fi).”.*
