# Supplementary material for: Determining the timing of pubertal onset via a multicohort analysis of growth
Source: PLoS One. 2021 Nov 18;16(11):e0260137. doi: 10.1371/journal.pone.0260137 (PMC8601458; doi:10.1371/journal.pone.0260137)
Supplement: Attachment — Submitted filename: Response to Reviewers.docx [file pone.0260137.s007.docx]

**Response to Reviewers**

**Reviewer comment:** The authors present SITAR analysis of growth relative to pubertal analysis using three Finnish cohort studies—the Type 1 Diabetes Prediction and Prevention (DIPP) Study, the Special Turku Coronary Risk Factor Intervention Project (STRIP), and the Boy cohort. The authors conclude that their novel modeling approach provides an opportunity to evaluate Tanner breast/genital stage–based pubertal onset timing in cohort studies including longitudinal data on growth but lacking pubertal follow-up.

The study represents an important contribution to puberty research but the authors may have overstated the importance of their findings on line 458 and 459 as 'immediate clinical relevance and applicability'. Replication of these results is other dataset is warranted before such a generalization can be made. Perhaps they could suggest that this method is promising and with replication in other cohorts ...

*Thank you for bringing this up again. We have now re-changed the sentence on lines 477-480 (in a manuscript with tracked changes) as “The determination of pubertal onset timing based on pubertal growth markers is promising method and with replication in other cohorts it could facilitate the determination of the timing in adolescent cohort studies more generally.”*
